# Lessons from bamboo‐eating pandas and their gut microbiome: Gut microbiome flow and applications

**DOI:** 10.1111/eva.12915

**Published:** 2020-01-13

**Authors:** Zheng Zhang, Ting Hu, Guoqing Lu, Lifeng Zhu

**Affiliations:** ^1^ College of Life Sciences Nanjing Normal University Nanjing China; ^2^ Department of Biology University of Nebraska Omaha Omaha NE USA

**Keywords:** applications, gut microbiome flow, instability, local adaptation, wildness

## Abstract

The giant panda is one of the most endangered mammals in the world, and many studies have revealed their evolutionary adaptation to the local environment (e.g., dietary cellulose and cyanide) on the evidences from population genetics and their gut microbiome. Here, based on the results of our analysis of the giant panda gut microbiome, we concluded that instability and resilience are the two primary characteristics of the giant panda gut microbiome. This basic information may have an impact on giant panda conservation, as well the management of other animal species.

## 1 INTRODUCTION

There are many studies on the evolution of animals and their gut microbiome, especially in humans (Ley, Lozupone, Hamady, Knight, & Gordon, 2008; Schloissnig et al., 2013; Yatsunenko et al., 2012). The gut microbiome plays important roles in animal nutrition, behavior, health, immunity, and development (Ezenwa, Gerardo, Inouye, Medina, & Xavier, 2012; Kinross, von Roon, Holmes, Darzi, & Nicholson, 2008; Lee & Hase, 2014; Ley, Hamady, et al., 2008; Qin et al., 2014). Many diseases (e.g., obesity, diabetes, and autistic disorder) are caused by specific gut microorganisms (Forslund et al., 2015; Kostic et al., 2015; Ley et al., 2005; Mulle, Sharp, & Cubells, 2013; Turnbaugh et al., 2009); therefore, a better understanding of the relationship between humans and the gut microbiome would be beneficial for human health and disease treatment. Beyond the application in the treatment of human disease, in recent years, some scientists have considered the application of the gut microbiome in the conservation of endangered animals (Bahrndorff, Alemu, Alemneh, & Lund Nielsen, 2016; Jiménez & Sommer, 2017; Redford, Segre, Salafsky, del Rio, & McAloose, 2012; Stumpf et al., 2016; Trevelline, Fontaine, Hartup, & Kohl, 2019; Wei et al., 2019; Yao, Xu, Lu, & Zhu, 2019). The scientists first propose the concept of conservation metagenomics, which is along with current methods, major scientific issues and significant implications in the study of host evolution, nutrition, physiology, and ecology and conservation (Wei et al., 2019). In addition, captivity was found to have a profound effect on gut microbiome community of Przewalski's horse (*Equus ferus przewalskii*) compared with that living in the wild habitat, and these results have important implications for conservation management of other endangered mammals (Metcalf et al., 2017). The gut microbiome of translocated populations of Père David's deer (*Elaphurus davidianu*) is enriched in some pathways related to putative sodium transport compared with those of captive populations, which may be potentially adapted to their high‐salt diet (Wang et al., 2019).

The giant panda is one of most endangered mammals in the world (Schaller, 1985), and many studies have revealed their evolutionary adaptation to the local environment (e.g., dietary cellulose and cyanide) on the evidences obtained from previous population genetics studies and research into their gut microbiome (Hu et al., 2017; Nie et al., 2019; Wei, Wang, & Wu, 2015; Zhao et al., 2013; Zhu, Wu, Dai, Zhang, & Wei, 2011; Zhu, Yang, et al., 2018). Giant pandas, belonging to the order Carnivora, are herbivorous, and their diet consists mainly of bamboo (Schaller, 1985). Many factors, such as diet and phylogeny, can influence animal gut microbial community (Ley, Hamady, et al., 2008). Thus, our group's first task was to investigate the relationship between the bamboo diet and gut microbiome of the pandas. We found some putative cellulose (main composition of the bamboo) digestion enzymes in their gut microbiomes, which may help the giant panda to digest the cellulose (Zhu et al., 2011). The prevalent microbial taxa found in the gut microbiome include Firmicutes and Proteobacteria (Zhu et al., 2011). However, when we further investigated the bamboo‐eating pandas across the wild and captive population levels, we discovered the unstable gut microbiome system (high variation in the abundance of Pseudomonadaceae and Clostridiaceae) under a similar diet (bamboo) (Yao, Yang, et al., 2019). Many studies suggest that a similar host diet will lead to a stable gut microbiome (Coyte, Schluter, & Foster, 2015; Lozupone, Stombaugh, Gordon, Jansson, & Knight, 2012). However, this hypothesis may not hold true in the bamboo‐eating pandas (Yao, Yang, et al., 2019). We speculate that the brief digestion time, short digestive tract, and fast intestinal peristalsis may lead to high concentrations of oxygen that select for the growth of aerobes and facultative anaerobes (e.g., Pseudomonadaceae from Proteobacteria) in giant pandas (Yao, Yang, et al., 2019). *The first lessons*: long‐term similarities in diet do not always lead to similar or stable gut microbial system within the same host species and other factors (e.g., host digestive system) can drive the selection of gut taxa.

The living environment, such as captivity, also has a profound effect on the animal gut microbial community. Our group previously revealed the difference in the gut microbiome between captive and wild populations (Yao, Xu, Hu, et al., 2019; Zhu et al., 2011). The causes of these differences may be complicated, including differences in dietary nutrition (e.g., different bamboo species), veterinary care, and sharing from humans. Considering the similar findings reported in many other animals (Clayton et al., 2016), this observed difference in the gut microbiome between captive and wild populations is not unexpected. However, based on long‐term monitoring of the gut microbiome of translocated giant pandas and local populations, we found the wildness of the gut microbiome of translocated pandas after their reintroduction into wild habitat (increasing in Pseudomonadaceae abundance, and enriching the pathways related to essential amino acid metabolic activity), and this process is neglected in the current translocation management (Yao, Xu, Hu, et al., 2019). Following translocation, the pandas from captive populations face competition from local wild pandas; thus, the wildness of behavior of the translocated panda may play a role in surviving in the local, wild habitat. However, there are some failed cases that resulted in the death of translocated individuals, which was caused by some bacterial pathogens after release into wild habitat. Here, we suggest that candidate pandas live with their mothers in a fenced area at the translocation site for an additional year prior to release to increase the putative evolutionary adaptation to the local environment at the translocation site (Yao, Xu, Hu, et al., 2019). *The second lesson*: we suggest that candidate pandas live with their mothers in a fenced area at the translocation site for an additional year prior to release. This is also more similar to the typical life cycle of the local wild giant pandas. Thus, this study will give an example of the applications of gut microbiome on the animal conservation and management.

In addition, our research on the endangered Père David's deer and its gut microbiome further reveals the local adaptation of translocated populations. For example, both the Père David's deer (positive selection genes related to blood pressure) and their gut microbiome are adapted to a high‐salt diet, and most of the coastal wetlands in China will be the potential translocation sites to resolve the saturation of current captive deer populations (Wang et al., 2019; Zhu, Deng, et al., 2018). Thus, *the third lesson*: combined with our findings in bamboo‐eating pandas, the resilience of animal gut microbiomes will provide important data regarding effective endangered animal management.

## 2 FUTURE DIRECTIONS

### 2.1 The longitudinal study on the giant panda gut microbiome among wild mountain populations

The giant panda gut microbial system is unstable, which may be caused by its special digestive system and the high concentration of bamboo metabolites (e.g., cyanide compounds) (Yao, Xu, Hu, et al., 2019; Zhu, Yang, et al., 2018). One study reveals that seasonal variation in nutrient utilization shape gut microbiome community and function in the wild giant pandas (Wu et al., 2017). Considering the putative seasonal changes in the nutritional source and secondary metabolites (e.g., tannins, and cyanides) in the bamboo diet, the longitudinal study of the giant panda gut microbiome (composition and function) will be one of the interesting questions among wild mountain populations. Multi‐omics approach (integrating metagenome, metatranscriptome, and metabolome) will provide the profiles on the interaction between microbial strains and ingredients of the bamboo diet.

### 2.2 Habitat protection and gut microbiome flow among fragmented populations

**Figure 1:**
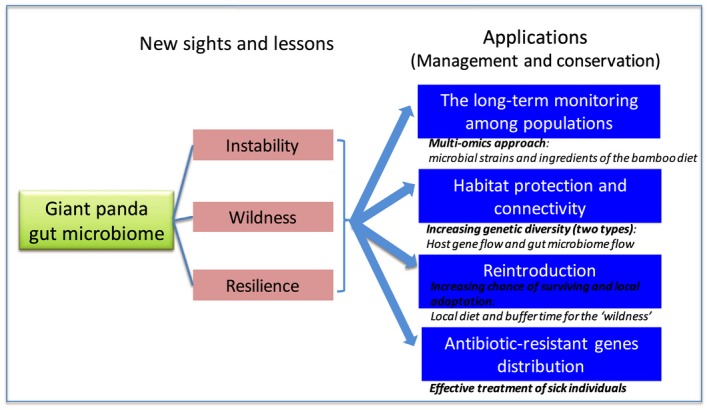
Giant panda gut microbiome and its potential application

In animal conservation, one of the most important strategies is to protect and recover animal habitat, which can help endangered species survive and breed. Additionally, increasing the connectivity among fragmented and isolated populations can increase gene flow and genetic diversity, which can mitigate negative effects due to inbreeding (Epps et al., 2005; Mech & Hallett, 2001). Giant pandas live in about 20 fragmented populations, and we currently do not have a detailed understanding of the panda gut microbiome on a large‐scale level. Habitat degradation has impacted the black howler monkey (*Alouatta pigra*) gut microbiome and resulted in the decrease of the alpha diversity of their gut microbiome (Amato et al., 2013). Other studies have revealed that the gut microbiome of the Udzungwa red colobus monkey (*Procolobus gordonorum*) in the undisturbed forest has significantly higher alpha diversity than that in the disturbed forest (Barelli et al., 2015). Thus, the evaluation of the relationship between fragmented habitat and gut microbiome diversity will be useful for the effective management of giant pandas. Gut microbiome sharing will happen both at the vertical level (from mother to offspring) and the horizontal level through the shared environment and social behavior (Moeller, Suzuki, Phifer‐Rixey, & Nachman, 2018). Currently, based on results in primate studies (Amato et al., 2013; Barelli et al., 2015) and the role of gut microbiome on the health of the host, we speculate that increasing the connectivity between individuals (pandas) of fragmented populations will increase the gut microbiome flow and diversity along with the host (panda) gene flow (Figure 1). However, this proposed model should be verified in the future. The wide impact of this scientific question is that habitat protection and recovery will increase either the gene flow of the host or that of the gut microbiome (symbiosis). These two types of flow may be beneficial to the overall fitness of the species due to mitigation of inbreeding effects and adaptation to local environment.

### 2.3 Animal gut microbiome and translocation

Based on our previous research, we found some differences in the gut microbiome between wild Xiaoxiangling population and Minshan populations (Yao, Xu, Hu, et al., 2019). Many captive giant pandas are the offspring from the wild pandas having the mountain origin information. Thus, both host genetic and gut microbiome backgrounds should be considered during the selection of candidate translocated individuals, which may increase the genetic and gut microbiome diversity. Moreover, wildness has been found in translocated animals (Yao, Xu, Hu, et al., 2019), and thus, the wildness process and period should also be considered in the future while reintroducing giant panda and other endangered animals (Figure 1).

### 2.4 Antibiotic‐resistant genes (ARGs) in the animal gut microbiome

Antibiotic‐resistant pathogens can have a profound effect on animal and human health (Allen et al., 2010; Zhu et al., 2013). Antibiotic‐resistant genes (e.g., aminoglycoside, glycylcycline, macrolide, beta‐lactam, puromycin, and bacitracin) are enriched in the captive panda gut microbiome compared with that of the wild pandas (Guo et al., 2019). However, we do not know the distribution of the ARGs at the large‐scale level across different captive and wild populations. This research will provide us with some basic information for the treatment of sick pandas; the ARG distribution of the gut microbiome will also help in assessing the ARG population within each captive center housing many different animals (e.g., zoos) (Figure 1).

## CONFLICT OF INTEREST

None declared.

## AUTHOR CONTRIBUTIONS

All authors contributed to the manuscript writing. ZZ and TH contributed equally to this work.

## Data Availability

Data sharing is not applicable to this article as no new data were created or analyzed in this study.
